# Epoxidation of Kraft Lignin as a Tool for Improving the Mechanical Properties of Epoxy Adhesive

**DOI:** 10.3390/molecules25112513

**Published:** 2020-05-28

**Authors:** Julia R. Gouveia, Guilherme E. S. Garcia, Leonardo Dalseno Antonino, Lara B. Tavares, Demetrio J. dos Santos

**Affiliations:** 1 Nanoscience and Advanced Materials Graduate Program (PPG-Nano), Federal University of ABC (UFABC), Santo André 09210-580, Brazil; juliargouveia@gmail.com (J.R.G.); guilherme.elias@outlook.com (G.E.S.G.); leonardo.dalseno@aluno.ufabc.edu.br (L.D.A.); lara.btavares@hotmail.com (L.B.T.); 2 Materials Engineering Graduate Program (PPG-Nano), Federal University of ABC (UFABC), Santo André 09210-580, Brazil

**Keywords:** bio-based adhesives, lignin-based epoxy

## Abstract

Owing to its chemical structure, wide availability and renewable nature, lignin is a promising candidate for the partial replacement of fossil-based raw material in the synthesis of epoxy resins. Its poor compatibility has been reported to be one of the main drawbacks in this domain. On the other hand, a well-established modification method for lignin epoxidation has been used for many years for the improvement of lignin compatibility. However, the extent of the effect of lignin epoxidation on the improvement of bio-based epoxy mechanical properties, applied as adhesives, is still an open question in the literature. In this context, a pristine and industrial grade kraft lignin (AKL) was reacted with epichlorohydrin to yield epoxidized lignin (E-AKL) in this work. Afterwards, AKL or E-AKL were separately blended with petroleum-based epoxy resin at 15 and 30 wt% and cured with a commercial amine. The adhesive curing kinetic was evaluated using a novel technique for thermal transition characterization, Temperature Modulated Optical Refractometry (TMOR); the results showed that the incorporation of AKL reduces the crosslinking rate, and that this effect is overcome by lignin modification. Mechanical tests revealed an improvement of impact and practical adhesion strength for samples containing 15 wt% of E-AKL. These results elucidate the effect of lignin epoxidation on the application of lignin-based epoxy adhesives, and might support the further development and application of these bio-based materials.

## 1. Introduction

Good mechanical and thermal properties, chemical resistance, and proper adhesion to many materials are some of the properties that explain why epoxy resins are among the widely most applied thermoset polymers [1,2]. This large and superior range of properties resulted in the application of a high volume of epoxy, as revealed by the market size. In North America, for example, epoxy resins are expected to generate $1.4 billion this year [3]. The large amount of epoxy consumption is explained by its application in several industrial segments, such as composite matrices, circuit boards, coatings, and adhesives. Particularly as adhesives, epoxy systems are the preferred materials for joining metals in aerospace, automobile, and civil engineering applications [4,5,6]. Although several types of epoxy resins are available, diglycidyl ether of bisphenol A (DGEBA) is by far the most used one. DGEBA is synthesized from bisphenol-A (BPA) and epichlorohydrin, in which BPA is usually responsible for up to 67% of the molar mass. Besides its broad industrial application, BPA has been proven to be highly toxic. Several studies have shown that BPA is an endocrine disrupting chemical (EDC), i.e., it can affect several kinds of receptors, including estrogen and androgen receptors [7]. Owing to the hazardous potential of BPA to living organisms, its application has been restricted in some countries [3,8].

**Figure 1:**
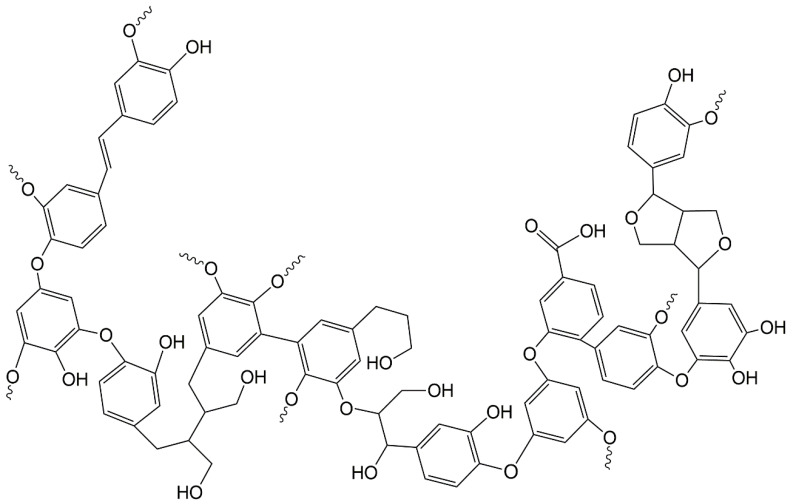
Lignin fragment hypothetical structure. Adapted from [22].

Consequently, the search for a substitute for BPA has motivated several research groups in the past few years. At the same time, the depletion of fossil raw materials and the growing interest in green and sustainable chemistry have drawn attention toward renewable precursors for bio-based polymers synthesis. In this context, research efforts have been dedicated to synthesizing bio-based epoxies, employing vegetable oils [9,10,11], tannin [12,13,14], rosin [15,16], and lignin. Among these, lignin might be expected to offer similar properties to conventional petroleum-based epoxy resins, due to its aromatic backbone structure and the high amount of phenolic hydroxyl moieties (Figure 1) [17]. Furthermore, technical grade lignin is generated in high volume as a byproduct from pulping and the paper industry, and does not compete with food crops, therefore representing an opportunity for upcycling [18,19,20,21].

**Figure 2:**
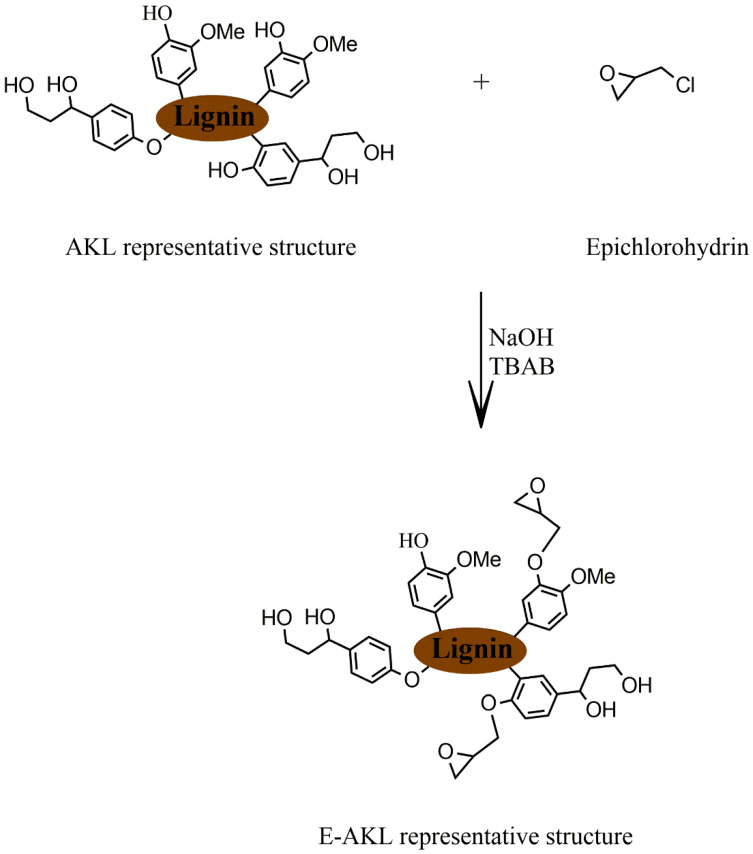
Epoxidation reaction scheme.

A literature review showed that there are three main methods for the development of lignin-based epoxy resins [17,19]: (1) direct incorporation of unmodified lignin, blending it with petroleum-based epoxy resins, (2) epoxidation of hydroxyl groups in the lignin structure, mainly through the reaction with epichlorohydrin, and (3) lignin epoxidation after prior derivatization. The first one is the easiest; however, unmodified lignin usually does not spontaneously react with epoxy and amine groups, and tends to agglomerate during epoxy curing. This method can generate brittle materials and impose a restriction on the maximum amount of lignin incorporation [23,24]. The second approach attempts to overcome these limitations and enhance the epoxy mechanical properties. Lignin epoxidation, through its reaction with epichlorohydrin (Figure 2), has been widely investigated in recent years, and has emerged as a feasible way to introduce epoxy groups into the structure, increasing its compatibility to that of petroleum-based epoxy resins and improving its mechanical properties. The third method is based on lignin chemical modification (e.g., phenolation [25], hydromethylation [26,27], or depolymerization [28]) prior to its reaction with epichlorohydrin.

Although previous lignin modification might improve lignin reactivity towards epichlorohydrin, this approach has the disadvantage of demanding additional reaction steps, which might involve hazardous/expensive reagents or complex processes (high pressure and/or temperature), increasing safety requirements, costs and hindering its industrial application.

In this work, we investigate the effects of lignin epoxidation on the mechanical properties of epoxy adhesives. On one hand, lignin epoxidation through its reaction towards epichlorohydrin is a well-known and established method. On the other hand, the effects of lignin epoxidation on the mechanical properties of epoxy adhesives are still understudied in the literature. Here, these effects were investigated using a large and industrially available, technological grade lignin. Unmodified and epoxidized lignin were blended with industrial epoxy resin. Afterwards, the relationship between lignin modification and lignin content and the mechanical properties of epoxy adhesives was established. Furthermore, a novel thermal analysis technique, i.e., Temperature Modulated Optical Refractometry (TMOR), was applied to investigate lignin-based epoxy curing. The results revealed the potential of epoxidized lignin to improve the impact and practical adhesion strengths of epoxy adhesives, and might support the development and application of these bio-based epoxy resins.

## 2. Experimental Procedures

### 2.1. Materials

Technical grade hardwood alkali Kraft lignin (AKL), a byproduct of pulp and paper production, was kindly provided by Suzano Papel e Celulose (Suzano, SP, Brazil) with the following characteristics: brown powder, pH 8.1, solid content = 92.5%, and hydroxyl index equivalent to 220 mg KOH·g^−1^, being around 60 mg KOH·g^−1^ from aliphatic-OH and 160 mgKOH g^−1^ from aromatic-OH (determined by ^31^P nuclear magnetic resonance, according to methodology described in [29,30]). Tetrabutylammonium bromide (TBAB) was purchased from Sigma-Aldrich Brazil (São Paulo, SP, Brazil). The petroleum-based epoxy resin (LR200) and crosslinker agent (LE20) were acquired from ABCOL (São Caetano do Sul, SP, Brazil). The epoxy resin was a low-viscosity liquid and had an epoxy index of 186.4 g/eq. The hardener is a cycloaliphatic diamine and had an amine equivalent weight of 186 g/eq. The other chemicals used in this study (epichlorohydrin and sodium hydroxide) were purchased from Labsynth (Diadema, SP, Brazil), and were used as received.

### 2.2. Lignin Epoxidation

Epoxidized alkali kraft lignin (E-AKL) was obtained by the reaction of AKL with epichlorohydrin (general reaction scheme shown in Figure 2). First, 40 g of AKL was dissolved in a mixture of 120 g of epichlorohydrin (at an epichlorohydrin/ALK ratio of 1:3 respectively) and 120 mL tetrabutylammonium bromide (TBAB, 2 wt% of AKL). The solution was charged into a 1000 mL three-neck, round-bottom reactor and heated to 60 °C under mechanical stirring. After temperature stabilization, 500 mL sodium hydroxide solution (NaOH 12%) was added dropwise into the reactor and maintained at 60 °C for 8 h. Then, the reaction mixture was vacuum-filtered, and washed several times with distilled water to remove the alkaline reagent. The solid residue was dried at 100 °C under vacuum for 24 h to remove the residual epichlorohydrin. Subsequently, the resulting powder was separated for characterization and used for the following reactions with DGEBA and the curing agent. Finally, the content of epoxy groups was determined by titration [31] and revealed an epoxidized lignin with 0.5% of epoxy index.

### 2.3. Epoxy Synthesis

Bio-based epoxy was obtained by mechanically mixing the commercial BPA-based epoxy resin (DGEBA) with AKL or E-AKL in percentages of 15/85 and 30/70 lignin/DGEBA. The curing agent was added according to epoxy index of DGEBA and epoxidized lignin. After the addition of the curing agent, the resin was cured at room temperature for 24 h and used to obtain the specimens from the other performed analyses. The conventional epoxy (i.e., petroleum based) is referred to as EPX. The lignin-based epoxy nomenclature indicates the type and quantity of lignin employed in the epoxy synthesis. EPX-15-AKL and EPX-30-AKL refer to samples with 15 wt% and 30 wt% AKL, respectively. Accordingly, EPX-15-E-AKL and EPX-30-E-AKL are the epoxidized-lignin counterpart.

### 2.4. Infrared Spectroscopy (FTIR)

Fourier transform infrared spectroscopy (FR-IR) was used to analyze the chemical structures of AKL and E-AKL. The FTIR spectra were collected on a Varian Agilent 640-IR FT-IR (Santa Clara, USA) in Attenuated Reflectance (ATR) mode, with 40 total scans, a wavenumber from 4000 cm^−1^ to 600 cm^−1^ and a resolution of 1 cm^−1^.

### 2.5. Differential Scanning Calorimetry (DSC)

The thermal behavior of nonmodified and epoxidized lignin were investigated by differential scanning calorimetry (DSC, Netzch DSC 204F1 Phoenix). Approximately 10 mg of sample was weighed in an aluminum pan and sealed by an aluminum lid. Initially, the sample was ramped to 135 °C at 10 °C min^−1^, kept at this temperature for more 30 min, and cooled to 35 °C at the same rate, in order to erase the thermal history (methodology adapted from [32]). Then, another heating cycle was performed at the same heating rate, and the thermogram was recorded for analysis.

### 2.6. Temperature Modulated Optical Refractometry (TMOR)

Temperature Modulated Optical Refractometry (TMOR) is a recently developed dilatometry technique based on optical refractometry. In this section, the basic theoretical background of the technique is presented. TMOR analysis is based on the acquisition of the refractive index N of the sample (liquid or solid). N is acquired in the total reflection of the light regime, which takes place at the contact between the sample and a high-refractive-index sapphire prism (for a more detailed description, the reader is referred to [33,34,35]). The measurement can be carried out isothermally or during a temperature ramp.

It is already well-established that N is closely related to the density (ρ); this relationship has been described by several models, with Lorentz-Lorenz (Equation (1)) being the most prominent one [33].

(1) $$\frac{N{\left(X\right)}^{2}-1}{N{\left(X\right)}^{2}+2}=r.\rho \left(X\right)$$

where N is the refractive index, ρ is the density, X is time or temperature, and r is the specific refraction. The latter quantity is related to the molecular polarizability, and can be assumed to be constant, as this quantity is weakly dependent on temperature. Considering that the sample mass is constant throughout the measurement, one can then assess the specific volume v (v=1/ρ) and the volume variation during temperature or time sweep:

(2) $$v\left(X\right)=\frac{N^{2}\left(X\right)+2}{N^{2}\left(X\right)-1}$$

The incremental change in volume can be calculated using Equation (3). If the measurement is carried out in a temperature ramp, Equation (3) will yield the volumetric expansion coefficient ($\beta_{q}\left(T\right))$ [33,36]. When performed isothermally, TMOR analysis might yield the shrinkage (β(t)) [37], which can be a consequence of polymerization and/or molecular rearrangement, as will be discussed later.

(3) $$\beta =-\frac{1}{\rho }\partial_{X}\rho =\frac{-6}{\left(N^{2}+2\right)\left(N^{2}-1\right)}\frac{\mathrm{dN}}{\mathrm{dX}}$$

Another key aspect of TMOR is its capacity to impose a sinusoidal temperature modulation on the sample during the measurement, as described in Equation (4).

(4) $$T\left(t\right)=\bar{T}+A^{T}\sin\left(2\pi ft\right)$$

where T(t) is the instantaneous temperature, $\bar{T}$ is the average temperature, $A^{T}$ is the modulation amplitude, and f is the modulation frequency. The small temperature perturbation described in Equation (4) induces a refractive index response which naturally follows the same sinusoidal profile, but with a phase shift between both signals, as presented in Equation (5) [38].

(5) $$n\left(t\right)=\bar{n}+A^{n}\sin\left(2\pi ft-\phi \right)$$

where n(t) is the instantaneous refractive index, $A^{n}$ is the refractive index amplitude, and $\bar{n}$ is the average response to $\bar{T}$, which will hereafter be referred as N_MEAN_. In accordance with the literature [33,39], the temperature modulation part described in Equations (4) and (5) yields complementary information about thermal expansion, namely the dynamic thermal volume expansion coefficient $\beta^{\prime }$ and $\beta^{\prime \prime }$ (Equations (6) and (7), respectively) [37].

(6) $$\beta^{\prime }\left(f,\bar{T}\right)=\left[6\bar{n}\left(\bar{T}\right)A^{n}\left(f,\bar{T}\right)\cos\left(\phi \left(f,\bar{T}\right)\right)]/[A^{T}\left({\bar{n}}^{2}\left(\bar{T}\right)+2\right)\left({\bar{n}}^{2}\left(\bar{T}\right)-1\right)\right]$$

(7) $$\beta^{\prime \prime }\left(f,\bar{T}\right)=\left[6\bar{n}\left(\bar{T}\right)A^{n}\left(f,\bar{T}\right)\sin\left(\phi \left(f,\bar{T}\right)\right)]/[A^{T}\left({\bar{n}}^{2}\left(\bar{T}\right)+2\right)\left({\bar{n}}^{2}\left(\bar{T}\right)-1\right)\right]$$

The acquisition of the dynamic thermal volume expansion coefficient might yield new information regarding possible relaxation processes due to the sinusoidal temperature perturbation, including during the early stages of cure. Identifying the occurrence of these processes, as well as the shrinkage behavior during adhesive curing, is of uttermost importance for the design of joints. To evaluate the effect of lignin and modified lignin on the epoxy curing behavior, as well as on the aforementioned parameters, TMOR was carried out on a thermo-optical oscillating refraction analyzer TORC 5000, from Anton Paar (Sao Paulo, Brazil). This equipment has an absolute refractive index accuracy of ca. 10^−6^ and a prism temperature accuracy of 10^−2^ °C. The homogenous reactive epoxy and lignin-based epoxy mixture was poured onto the crystal of the TMOR cavity immediately after its synthesis. The curing process was monitored at an average temperature of 25 °C with a modulation period of 60 s and temperature amplitude of ±0.5 °C for 12 h. The average refractive index (N_MEAN_) and the dynamic thermal volume expansion coefficients ($\beta^{\prime }$ and $\beta^{\prime \prime }$) were recorded for the entire period, and the shrinkage coefficient (β(t)) was calculated according to Equation (3).

### 2.7. Izod Impact Test

The Izod impact strength was determined according to ASTM D 256, test method A and nominal pendulum energy capacity of 2.7 J, using Shanta Engineering equipment. Notched specimens presented the following dimensions: 65 mm × 12.70 mm × 3.5 mm. Five samples were tested for each epoxy composition.

### 2.8. Single-Lap Shear Test

Joint strength was determined by single lap shear test following the ASTM D1002-01 standard. The adhesive reactive mixture was placed at both surfaces of steel plates (100.0 × 25.4 × 1.5 mm), which were then assembled into single-lap shear samples and kept together by grips for 7 days. The adhesive thickness was 0.20 ± 0.02 mm and the dimensions of the overlapped joints were 25.4 × 12.7 mm. Five samples were tested for each adhesive composition.

## 3. Results and Discussion

This section is divided into two parts: The first discusses lignin chemical modification and its effects on chemical properties; The second part is related to the characterization of AKL- and E-AKL-based epoxy systems and their application as adhesives.

### 3.1. Characterization of Modified Lignin

#### 3.1.1. FTIR-ATR

**Figure 3:**
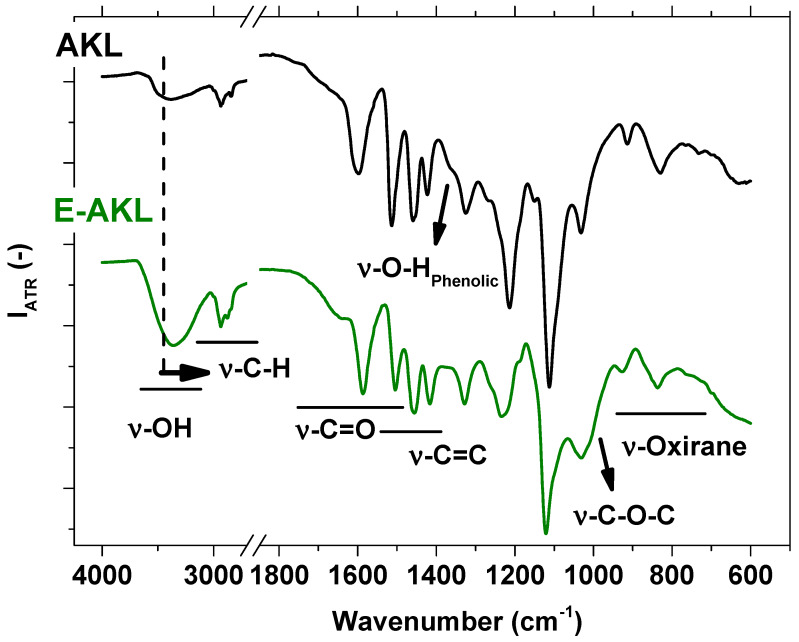
FTIR-ATR spectra of lignin as AKL (top) and E-AKL (bottom) in the 4000–650 cm^−1^ wavenumber region.

Figure 3 shows the AKL (top) and E-AKL (bottom) FTIR-ATR spectra, which may be used to identify changes in lignin chemical state after its reaction with epichlorohydrin, highlighting the main lignin- and epoxy-related bands.

The lignin spectrum is mainly characterized by a broad -OH band (centered around 3400 cm^−1^) and intense bands centered at 2935 cm^−1^ and 2841 cm^−1^, assigned to -CH_2_/-CH_3_ asymmetrical and symmetrical stretching, respectively. In addition, there is a low intensity -C=O stretching vibration at 1712 cm^−1^ and a stronger one located at 1600 cm^−1^, which is a combination of -C=O and -C=C from aromatic ring vibration. Finally, there is the characteristic aromatic skeleton -C=C vibration at 1515 cm^−1^.

A comparison between epoxidized lignin and unmodified lignin spectra revealed some indications of chemical differences between samples. The first evidence of chemical modification is the slight shift of the hydroxyl band, from 3400 cm^−1^ to 3300 cm^−1^, in agreement with [28] and [40]. In addition, a new peak appeared at 2873 cm^−1^, which can be assigned to the C−H stretching vibration, confirming that the lignin structure was modified as a consequence of the reaction with epichlorohydrin [41]. The shoulder located at around 1370 cm^−1^, which is assigned to aromatic -OH vibration, can no longer be seen in the E-AKL spectrum, suggesting the functionalization of these moieties with epichlorohydrin. Unfortunately, clear identification of the peaks related to the oxirane ring was not possible in this work due to the overlapping with nonmodified lignin peaks. However, a comparison between both spectra reveals some characteristic features that indicate the occurrence of an epoxidation reaction. The peak located at 1030 cm^−1^, which is assigned to ether bonds, shows significant broadening after reaction, suggesting the inclusion of an epoxy ring [31,42,43]. Furthermore, a slight broadening of the peaks located at 920 cm^−1^ and 835 cm^−1^ suggests the appearance of peaks that overlap with the previous ones. Both wavenumbers (920 cm^−1^ and 835 cm^−1^) are assigned to the oxirane ring [42,44].

#### 3.1.2. Differential Scanning Calorimetry

**Figure 4:**
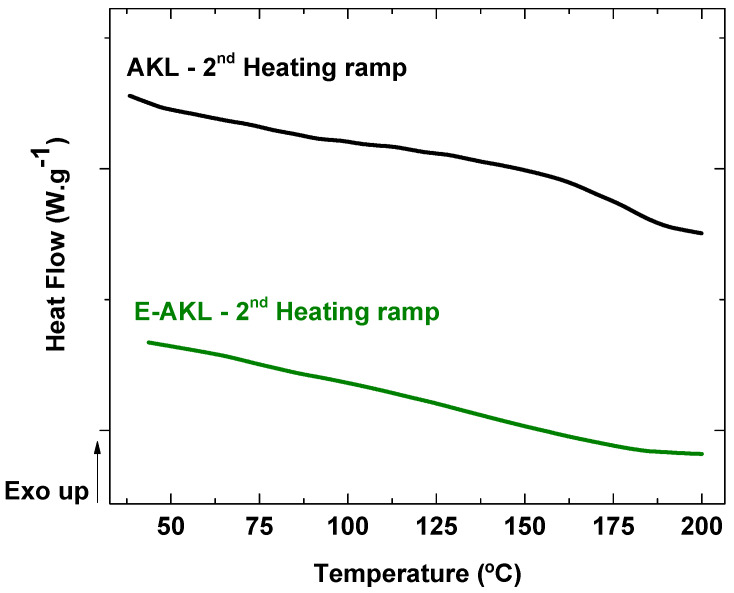
Heat flow (W·g^−1^) DSC curves obtained during the second heating scan of AKL and E-AKL.

Figure 4 shows the second-heating scan curve of unmodified lignin and epoxidized lignin. The AKL curve presented a step-like decrease in the heat flow which is assigned to glass transition. The glass transition temperature (T_g_) was identified as the midpoint of the step-down, and is T_g_ = 169 °C. The E-AKL curve, however, showed no thermal event in the investigated temperature range. The absence of any thermal event after epoxidation confirmed molecular changes in the lignin structure, but followed an unexpected pattern. Lignin modification by reaction with epichlorohydrin should result in the reduction of the lignin glass transition temperature, since this reaction causes (1) an increase in the overall molecule free volume (-OH volume < -C_2_H_3_O volume), and (2) a reduction in intermolecular interactions (less hydrogen bonding).

**Figure 5:**
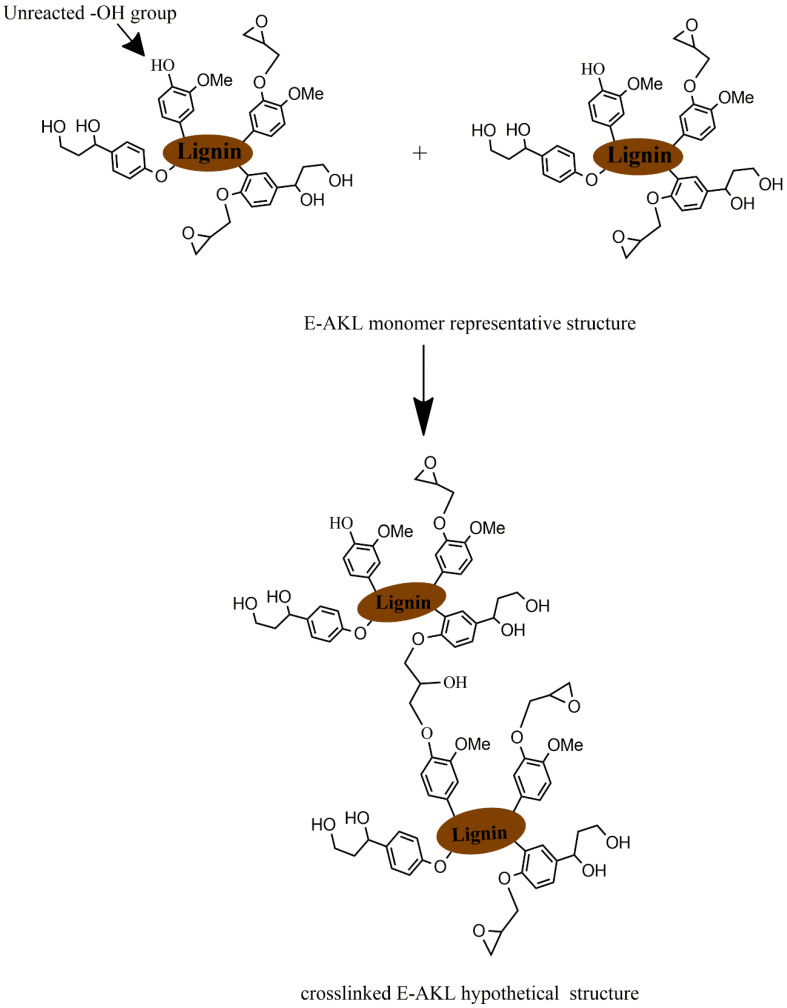
Schematic of the possible cross-linking reaction between remaining lignin -OH moieties and the inserted epoxy groups.

According to [32,45], lignin is unstable above T_g_ because of radical coupling reactions involving the phenolic hydroxyl groups, leading to a crosslinked structure that leads to an increase in its molecular mass. Combining higher lignin reactivity and molecular mobility at temperatures close to T_g_, this unforeseen result might be explained by the occurrence of crosslinking during the first heating ramp, that could enhance the M_w_ and, consequently, the T_g_ of the epoxidized lignin. In this context, we propose that the newly inserted epoxide groups in the lignin structure reacted with the remaining hydroxyl moieties in an etherification reaction (for the detailed reaction mechanism, the reader is referred to [46]). In fact, several studies have employed hydroxyl-terminated polymers or monomers as cross-linking agents for epoxy resins, strengthening our argument [47,48,49,50,51]. Furthermore, the self-condensation of epoxidized lignin involving the remaining -OH groups in lignin structure has been proposed elsewhere [42]. The elevated temperature reached during the first heating scan may cause the onset of the thermal event at higher temperatures, i.e., perhaps even higher than the degradation temperature of lignin, due to the formation of irreversible cross-linking, as shown schematically in Figure 5.

### 3.2. Lignin-Based Epoxy Adhesives Characterization

#### 3.2.1. Temperature Modulated Optical Refractometry (TMOR)

**Figure 6:**
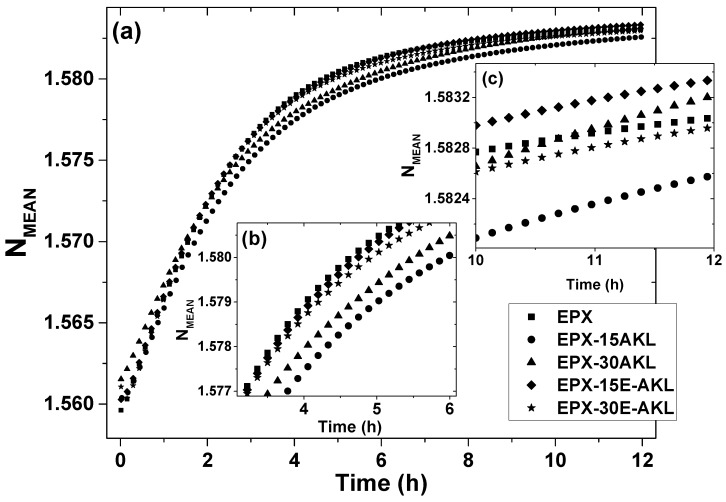
Temporal evolution of (**a**) the average refractive index (N_MEAN_) of all samples. Inset: (**b**) A zoomed-in representation of the early stages of the reaction (between 3 and 6 h). And (**c**) a zoomed-in representation of the final region (between 10 and 12 h).

TMOR analysis was carried out to investigate the early stage of EPX and lignin-based EPX cure. Figure 6a shows the temporal evolution of N_MEAN_ at 25 °C for all samples. The increase of N_MEAN_ mirrors the degree of chemical conversion during the isothermal polymerization of the lignin-based epoxies [38,52]. As the reaction proceeds, there is an increase in the ρ of the samples (according to Equation (1)) which can only be a consequence of the formation of a crosslinked network, thus, confirming the occurrence of a polymerization reaction. Additionally, the curves suggest that in the 12 h period, the reaction does not come to an end, since a plateau in the N_MEAN_ values was not reached.

**Figure 7:**
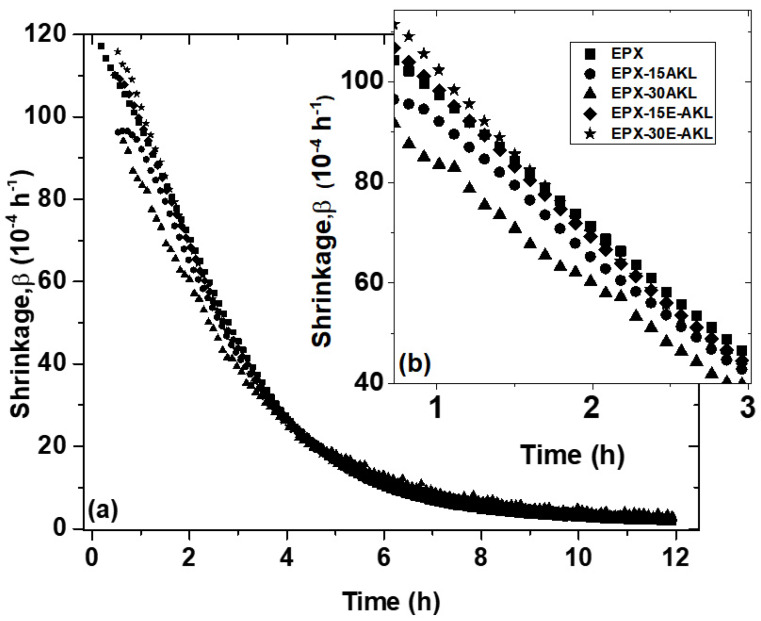
(**a**) Temporal evolution of the shrinkage coefficient (b, 10^−4^·h^−1^) of all samples. Inset: (**b**) A zoomed-in representation of the early stages of the reaction (between 1 and 3 h).

Although all curves show a similar trend, some key aspects illustrate the differences between the curing kinetic of the epoxy samples, particularly considering the outstanding accuracy of the N_MEAN_ measurement of 10^−6^. To investigate these subtle differences, the inclination of the curves was compared, as they mirror the reaction rate. For that reason, a fitting procedure was done in the Origin 2016 graphing software (option “QuickFit”). For Figure 6b, a quadratic function was used, while Figure 6c and Figure 7b were fitted by a linear function (r^2^ > 0.99 and reduce-chi^2^ < 0.1). All the fitted curves and the fitting parameters are displayed in the Supplementary Information (SI) file. In the early stages (0–3 h), the N_MEAN_ curves do not show any significant differences (this stage will be discussed in the context with Figure 7); however, as the reaction proceeds (Figure 6b), the pristine EPX and the samples with modified lignin (EPX-15-E-AKL and EPX-E-30AKL) show a higher reaction rate in comparison to the samples with nonmodified lignin (EPX-15AKL and EPX-30AKL), as illustrated by the steeper inclination of the N_MEAN_ curves. In the final stage (between 10 and 12 h, Figure 6c), there is an inversion on this pattern. At this point, EPX-15AKL and EPX-30AKL present a higher reaction rate (the inversion between EPX and EPX-15AKL happens at around 11 h), and the two formulations with modified-lignin show a lower reaction rate.

Figure 7 shows the shrinkage coefficient curve with respect to time. The shrinkage coefficient is an important parameter when designing bonded joints. The higher reaction rate for samples EPX, EPX-15-E-AKL, and EPX-30-E-AKL is also seen here, as highlighted in Figure 7b (see the fitted curves in SI). This outcome corroborates the evidence from Figure 6b that suggests a decrease in the curing rate with the incorporation of pristine lignin. In the last 2 h of the reaction, the shrinkage coefficient approaches zero, indicating that, at this point, there is no significant change in the sample volume.

**Figure 8:**
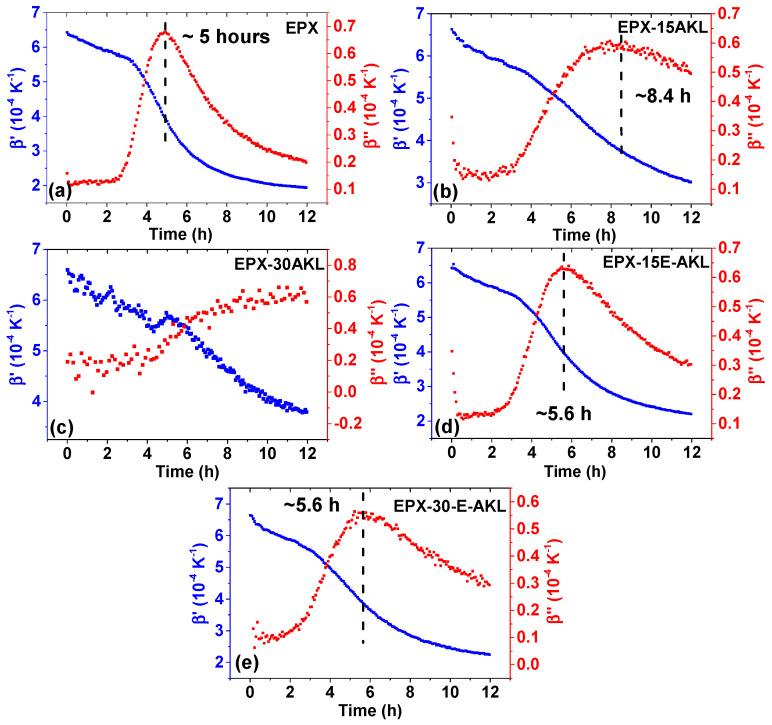
Temporal evolution of the dynamic thermal volume expansion coefficient of all samples (real part, b’, left axis and imaginary part, b’’, right axis). The chemically induced glass transition of each sample is indicated in the corresponding graph. (**a**) EPX, (**b**) EPX-15AKL, (**c**) EPX-30AKL, (**d**) EPX-15E-AKL, and (**e**) EPX-30-E-AKL.

As mentioned in the experimental section, TMOR is also able to impose a temperature modulation during the measurement that makes it possible to investigate the dynamic behavior of the samples. The evolution of the dynamic volume expansion coefficient is shown in Figure 8; it reveals some information that supports the previous discussion.

Figure 8a shows the behavior of β’ and β’’ over time for EPX. Starting at around 6 × 10^−4^·K^−1^, β’ shows a step-like decrease and then reaches a value of around 1.5 × 10^−4^·K^−1^. This behavior is accompanied by a peak in the β’’ curve that corresponds to the midpoint of the step transition seen in the β’ curve. The behavior of both dynamic volume expansion coefficients is typical for a volume relaxation, and considering that the final value of β’ (around 1.5 × 10^−4^·K^−1^) is typical of a polymer glass [53], it is reasonable to assume that the relaxation probed by TMOR is the so-called chemically induced glass transition, which happens at around 5 h. The chemically induced glass transition is assessed when the temperature is kept constant during the measurement, but due to a chemical process, the glass transition temperature of the sample increases to the temperature of the measurement, leading to vitrification [33]. The occurrence of vitrification in the sample is known to slow the reaction due to molecular mobility hindrance [37]. This topic has been addressed in a previous report from our group concerning the same epoxy system used in this work [38].

The addition of lignin to the epoxy system affects the behavior of the probed dynamic quantities. Figure 8b,c show the curves of EPX-15-AKL and EPX-30-AKL, respectively. At a lower lignin content (15%), the chemically induced glass transition starts, but it does not come to an end (β’ > 2 × 10^−4^·K^−1^ at the end of the measurement). Nevertheless, it is possible to estimate the time for its occurrence, i.e., around 8.5 h. With increasing the lignin content, there is no clear indication of the occurrence of vitrification in the investigated time. In fact, at the end of the measurement, β’ is even higher than 3 × 10^−4^·K^−1^, which confirms that the material is not in a glassy state. The delay in EPX-15-AKL vitrification and the absence of it in the EPX-30-AKL curve are indications that the addition of nonmodified lignin decreases the reaction rate, as suggested by both the N_MEAN_ and β curves. Additionally, this also explains the higher reactivity in the final stage of the measurement compared to EPX. As stressed above, EPX is already in a vitrified state, which hinders molecules mobility and reduces the reaction rate.

Figure 8c,d show the same set of plots for the modified lignin-based epoxy systems. Both 15 and 30% lignin content formulations present chemically induced glass transition. Additionally, the time at which the relaxation happens is not significantly affected by the inclusion of lignin, as both samples reach a vitrified state at around 5.6 h, in contrast to 5 h for the pristine epoxy. However, the shape of the β’’ curve clearly broadens with the inclusion of lignin, which might be related to the increase in heterogeneity due to the complexity of the structure of lignin. Nevertheless, this result indicates that in contrast to nonmodified lignin, the epoxidized counterpart does not severely impact the curing behavior of the epoxy resin, which is an indication of a higher reactivity towards the curing agent, and probably a consequence of reducing the steric hindrance of the reactive groups.

Finally, one last remark must be made concerning the contrast between the static and dynamic quantities (N_MEAN_, β vs β’ and β’’) of EPX and modified lignin-based-EPX. According to the dynamic curves, the three samples are in the glassy state after 10 h reaction time. Comparing the final stage of the N_MEAN_ curve, the EPX curve shows a higher reaction rate, since the curve is slightly steeper between 10 and 12 h of reaction time in comparison to EPX-15-E-AKL and EPX-30-E-AKL. This contrast indicates the complex and rigid lignin structure composed of several aromatic rings that hinder molecular mobility when in a glassy state.

#### 3.2.2. IZOD Impact Test

**Figure 9:**
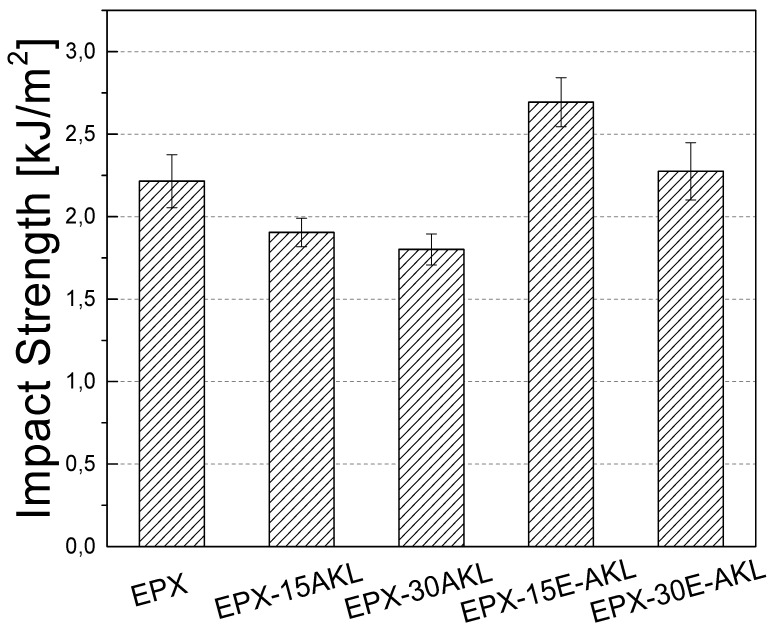
Impact strength of pristine and lignin-based epoxy.

Figure 9 displays the impact tests for each condition of the lignin-based epoxy resins. As shown, the neat epoxy (EPX) presented an impact strength of 2.20 kJ/m^2^, which decreased by the incorporation of unmodified lignin. Samples with 15 and 30 wt% unmodified lignin showed impact strengths of 1.85 and 1.70 kJ/m^2^, respectively.

There are other works in the literature in which it has been reported that the incorporation of lignin in epoxy matrices causes a reduction in impact resistance which is more pronounced in conditions of high lignin concentration [24,54]. The reduction of approximately 15 and 20% in the impact strength might be a consequence of the poor interaction between the unmodified lignin particles and the neat epoxy resin matrix [55].

On the other hand, the results showed that the incorporation of epoxidized lignin increased the impact strength of the cured epoxy. This increase is greatest for the EPX-15-E-AKL sample (2.70 kJ·m^2^). EPX-30-E-AKL presented a slightly higher impact strength than that of the epoxy resin (2.20 kJ·m^2^). The impact strength improvement of epoxidized lignin-containing epoxies may be attributable to the higher reactivity of the modified lignin, due to the incorporation of the epoxy groups in the lignin structure. This result agrees with our TMOR analysis, that suggested improved reactivity of the modified lignin-based EPX in comparison to its counterpart, i.e., amine groups (hardening). The formed covalent bonds improved the load transfer between the modified lignin and the neat epoxy, resulting in an increase of impact strength. Additionally, it is well-known that lignin epoxidation improves lignin dispersion in epoxy resins matrices, making it possible to incorporate higher amounts of lignin without compromising the mechanical behavior.

#### 3.2.3. Lap Shear Test

**Figure 10:**
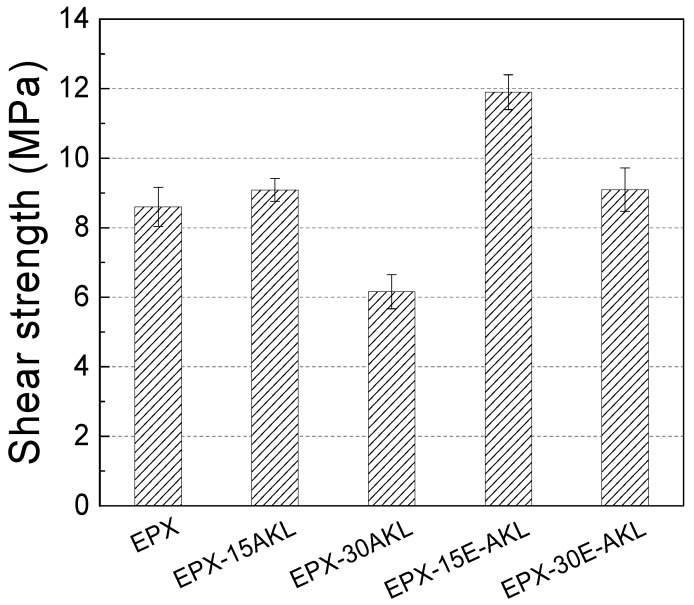
Single lap shear strength of the neat epoxy, nonmodified lignin-based epoxy, and epoxidized lignin-based epoxy in steel substrate.

Single lap shear steel specimens were obtained with neat epoxy and lignin-based epoxy adhesives. All samples showed cohesive failure after tests; the average shear strengths are presented in Figure 10. In general, the shear strength of adhesively bonded joints (practical adhesion [52,56,57]) was affected by lignin incorporation. Samples with 15 wt% of unmodified lignin were a unique condition which presented similar shear strengths in comparison to neat epoxy. Afterwards, the incorporation of higher amounts of unmodified lignin (30 wt%) strongly reduced this mechanical property; the same trend was observed in the impact tests. The negative influence of unmodified lignin in the practical adhesion of epoxy adhesives is probably a consequence of the low compatibility between DGEBA and the lignin at room temperature (curing temperature) that reduces the curing rate, as shown in the TMOR results. The unreacted lignin might form agglomerates which act like potential stress concentrators, decreasing the shear strength. This trend has been noticed in several works in the literature [24,58,59,60,61,62,63,64], and agrees with the impact test results.

Unlike unmodified lignin, lignin epoxidation provided a significant improvement of practical adhesion. Samples with 15 wt% epoxidized lignin increased their shear strength to 12 MPa, i.e., 39% higher in comparison with neat epoxy, while the sample with 30 wt% presented only a modest increase in shear strength in comparison to the pristine epoxy. The best practical adhesion observed for these samples is directly connected with the lignin modification that, as demonstrated previously, increased lignin reactivity and improved the resin mechanical properties. As the amount of modified lignin increases, there is a decrease in the shear strength. This is possibly a consequence of increased heterogeneity due to the complex structure of lignin, and it is an indication that 30 wt% is near the upper limit for lignin inclusion, which is in agreement with the findings in other works [24,43,65].

## 4. Conclusions

Kraft lignin was chemically modified by reaction with epichlorohydrin. The FTIR-ATR results confirmed the inclusion of oxirane rings in the structure of lignin, and suggested that aromatic -OH was the preferred target for epoxidation. A DSC analysis showed that after chemical derivatization, E-AKL showed no glass transition, which was attributed to the occurrence of self-condensation through the reaction between the newly-inserted epoxy rings and the remaining -OH groups on the lignin structure. This outcome is an indication of the relatively low thermal instability of the chemically modified lignin. TMOR analysis was employed for the first time as a tool to accompany the early stages of lignin-based epoxy resins. The results showed that the inclusion of AKL reduced the reaction rate, and that this reduction was proportional to the lignin content. In contrast, the addition of E-AKL did not significantly affect the reaction rate. This result provides a strong evidence that lignin modification increases its reactivity towards the hardener. The contrast on AKL and E-AKL reactivity was mirrored in the impact test and lap-shear results. While the use of AKL had a detrimental effect on the impact strength in comparison to pristine epoxy, the use of E-AKL, even at a higher concentration, showed the same effect. A similar trend was observed in the adhesive properties. At a low concentration of unmodified lignin (15 wt%), the mechanical behavior of the adhesively bonded joints was not compromised; however, with increasing the lignin content to 30 wt%, there was a decrease in the shear stress. In contrast, the addition of epoxidized lignin improved the mechanical properties, even at 30 wt%, making it possible to incorporate higher contents of lignin in epoxy resins. Thus, the effect of lignin modification on the curing behavior of lignin-based epoxy resin was demonstrated. These results might contribute to the reduction in the use of petroleum-based resins, along with the revalorization of a nonedible industrial waste that is generated in high amounts.

## Supplementary Materials

Supplementary Materials can be found online.
