# Supplementary material for: Epoxidation of Kraft Lignin as a Tool for Improving the Mechanical Properties of Epoxy Adhesive
Source: Molecules. 2020 May 28;25(11):2513. doi: 10.3390/molecules25112513 (PMC7321300; doi:10.3390/molecules25112513)
Supplement: Supplementary file 1 [file molecules-25-02513-s001.pdf]

Supplementary Information:

Fitting of all curves from Figure6 b:

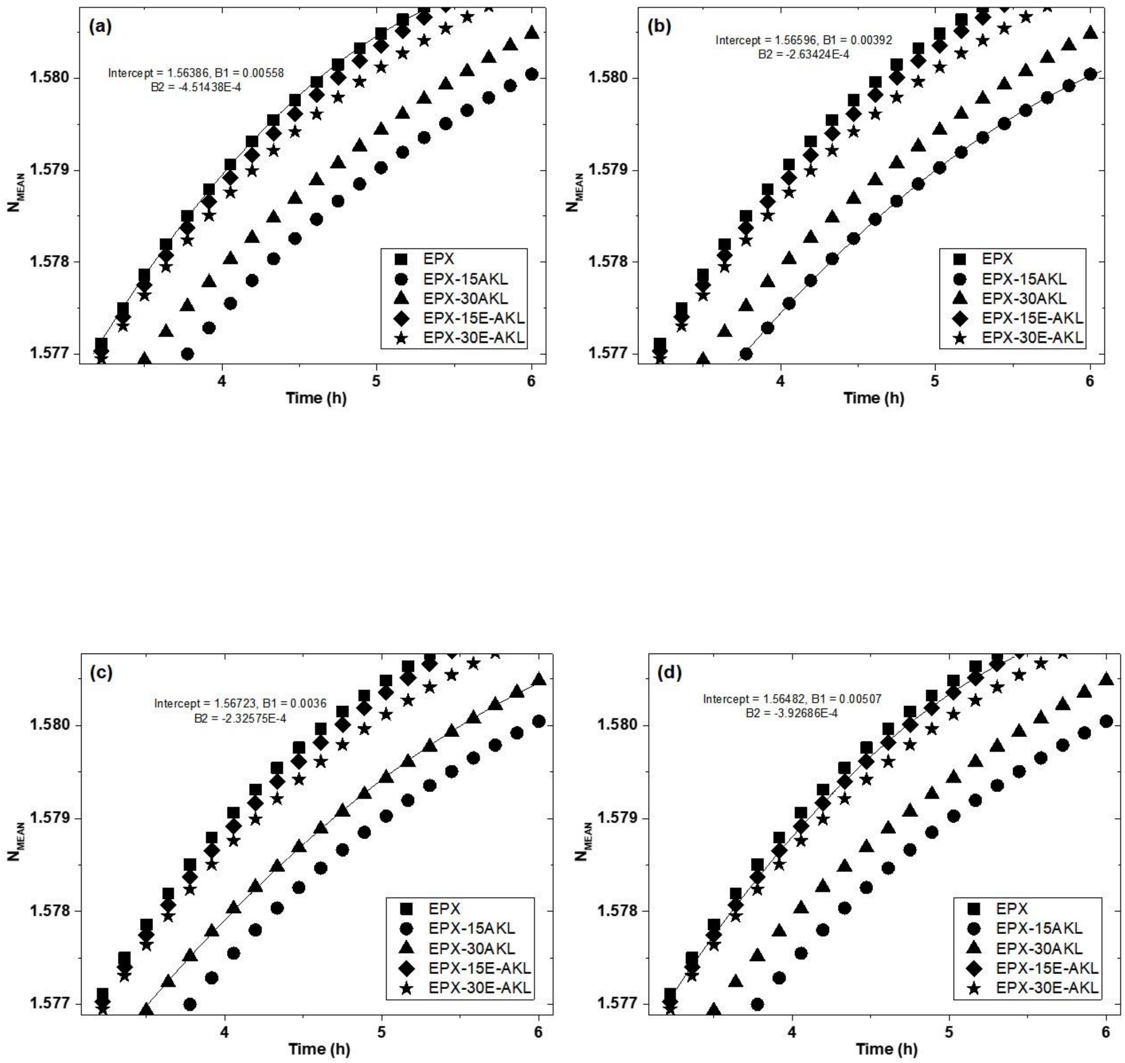

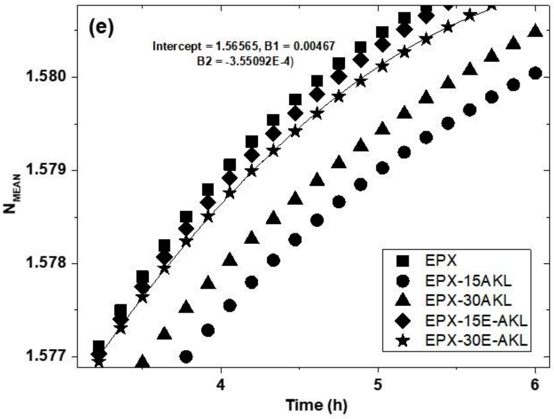

Fitting of all curves from Figure6 c:

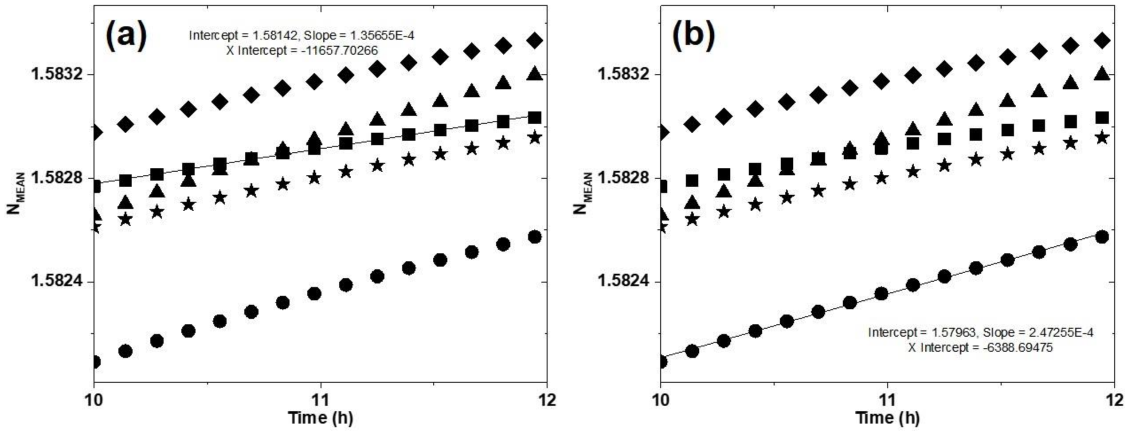

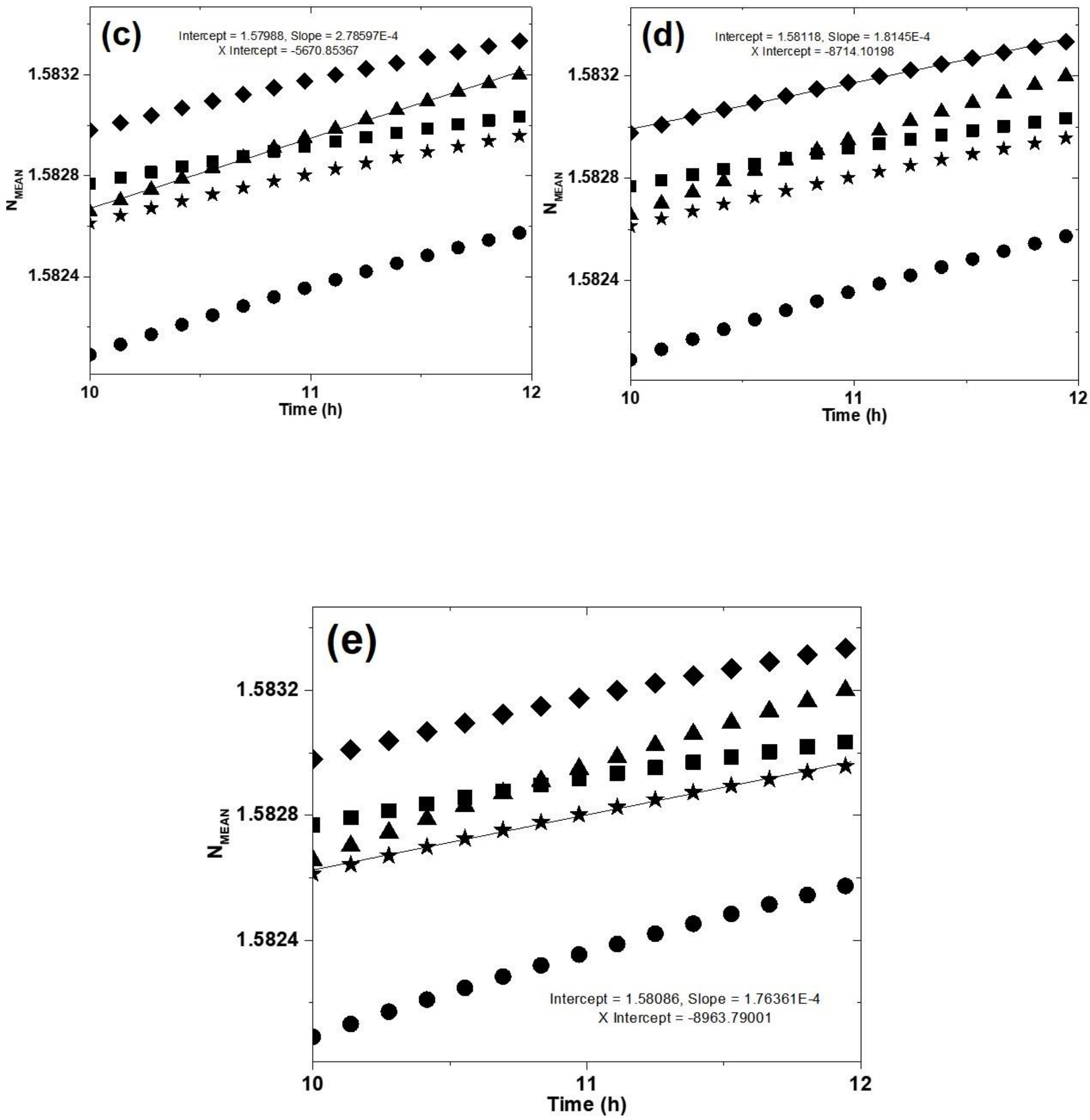
(c)
Intercept = 1.57988, Slope = 2.78597E-4
X Intercept = -5670.85367
N_MEAN
1.5832
1.5828
1.5824
10
11
12
Time (h)
(d)
Intercept = 1.58118, Slope = 1.8145E-4
X Intercept = -8714.10198
N_MEAN
1.5832
1.5828
1.5824
10
11
12
Time (h)
(e)
N_MEAN
1.5832
1.5828
1.5824
Intercept = 1.58086, Slope = 1.76361E-4
X Intercept = -8963.79001
10
11
12
Time (h)

Fitting of all curves from Figure7 b:

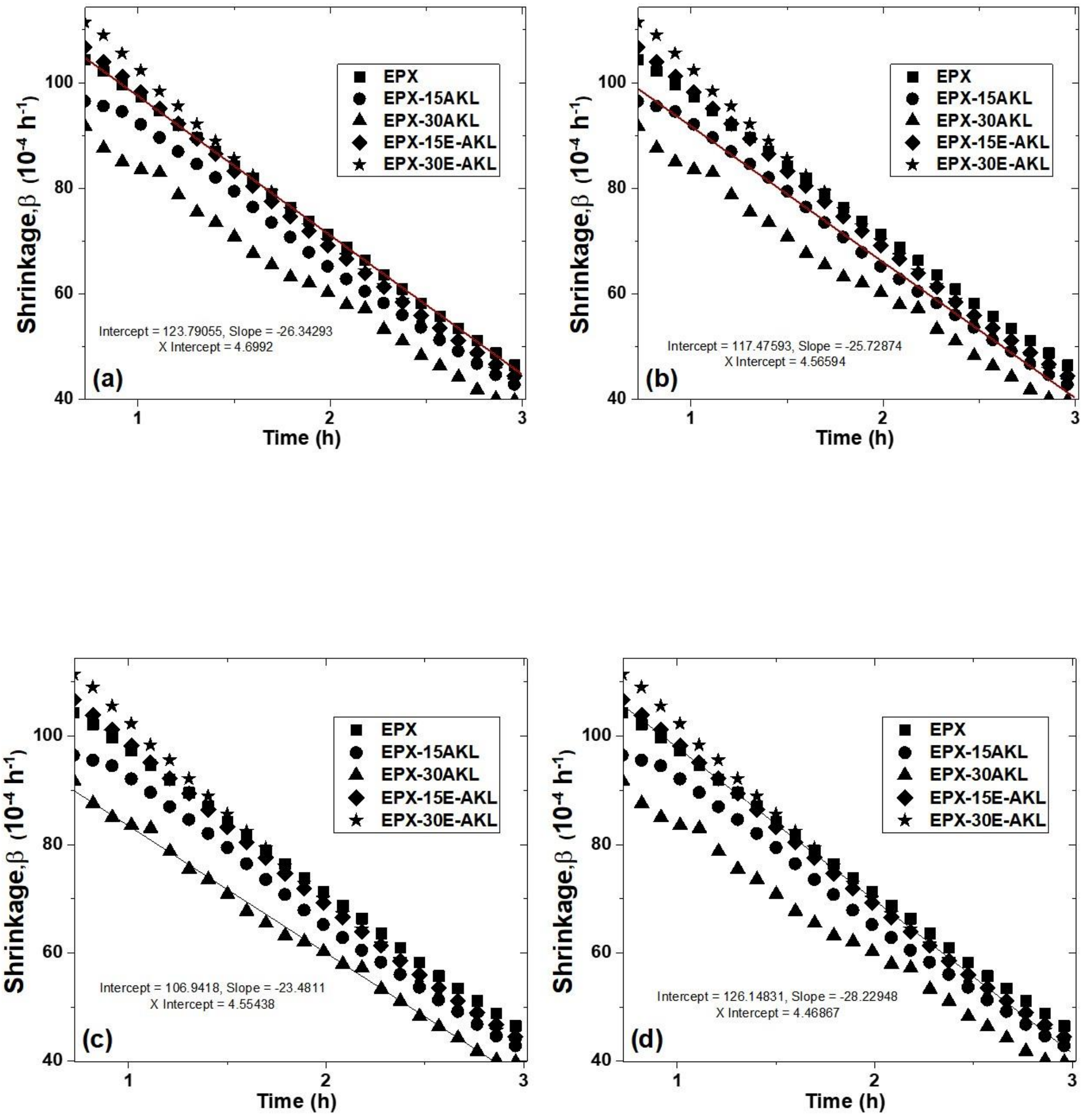

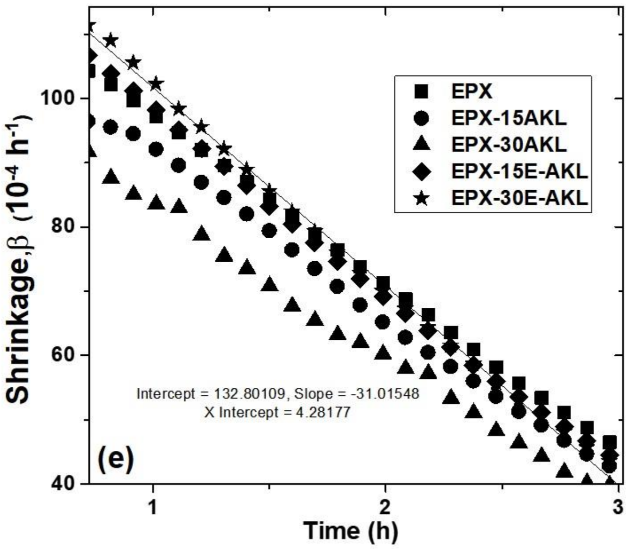

Shrinkage,β (10⁻⁴ h⁻¹)
Time (h)
EPX
EPX-15AKL
EPX-30AKL
EPX-15E-AKL
EPX-30E-AKL
Intercept = 132.80109, Slope = -31.01548
X Intercept = 4.28177
(e)
